# Lyotropic “Salty” Tuning for Straightforward Diversification and Anisotropy in Hydrogel Actuators

**DOI:** 10.1021/acs.langmuir.4c03291

**Published:** 2025-01-01

**Authors:** Pedram Tootoonchian, Levent Bahçeci, Andriy Budnyk, Halil I. Okur, Bilge Baytekin

**Affiliations:** † Chemistry Department, Bilkent University, Ankara 06800, Turkey; ‡ UNAM − National Nanotechnology Research Center, Bilkent University, Ankara 06800, Turkey

## Abstract

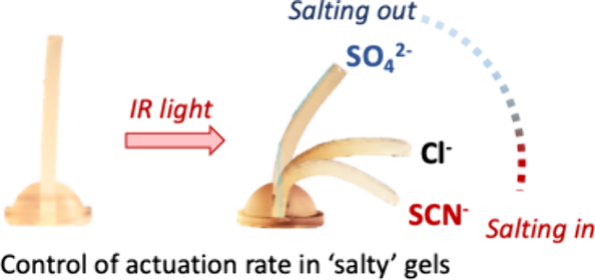

The specific ion effect (SIE), the control of polymer solubility in aqueous solutions by the added ions, has been a phenomenon known for more than a century. The seemingly simple nature of the ion–polymer–water interactions can lead to complex behaviors, which have also been exploited in many applications in biochemistry, electrochemistry, and energy harvesting. Here, we show an emerging diversification of actuation behaviors in “salty” hydrogel and hydrogel-paper actuators. SIE controls not only the dehydration speeds but also the water diffusion and mechanical properties of the gels, leading to composite actuation behavior. Most reported thermally activated hydrogel actuators suffer from expensive precursors or complex fabrication processes. This work addresses these issues by using a physicochemical effect displayed within an inexpensive gel with common salts. SIE-controlled anisotropic actuation in geometrically different systems provides a demonstration of how such physicochemical effects can lead to higher complexity in basic soft material design and hydrogel soft robotics.

## Introduction

Aqueous chemistry reigns in the metabolism of living organisms. All biochemical processes, e.g., transport, signaling, replication, and respiration, happen in an aqueous environment through delicate interactions of different species. The interaction of ions and large solutes like proteins control transport across membranes,^1^ molecular forces,^2^ protein function,^3^ enzyme activity,^4^ and growth.^5^ The ions affect the solubility and stability (precipitation) of polymers (proteins) and the dynamics and structure of water surrounding the polymer.^6−8^ The outcome of these polymer–ion–water interactions depends on the identity and concentration of the ions. First reported by Franz Hofmeister in 1888,^9,10^ and later generalized as “lyotropy” or “specific ion effects” (SIEs),^11,12^ the different behaviors of ions affecting physicochemical phenomena have been known for more than a century. Despite the long history of research on these interactions and their wide impact and importance in biochemical processes, the exact nature of these interactions covering different types of ions is still not completely known due to the involvement of many parameters of ion chemistry.^13^ In general, ions that can precipitate polymers by bringing order to the structure of water are called salting**-**out, and on the contrary, ions that enhance the solubility of the polymer (solute) by interacting with the backbone of the polymer, thus creating more solute–solvent interactions, are known as salting**-**in.^14−16^ Numerous (industrial) material applications, such as batteries,^17,18^ supercapacitors,^19^ metal electrodeposition,^20^ semiconductors,^21^ and fuel cells,^22^ utilize SIEs. In this report, however, we show that SIEs can be used in another field: soft robotics.^23^ Hydrogel actuators in soft robots can benefit from the “fine-tuning” of intermolecular interactions provided by SIEs.

The source of inspiration for water-controlled actuation in soft robotics and artificial systems is the water transfer mechanisms in plants.^24^ Transpiration, the transport of water from the soil through roots and stems, and evaporation from leaves are two of these mechanisms. Transpiration accounts for more than ca. 90% of the water used by plants and is vital for plants to adjust the temperature and osmosis. Osmosis, the water transport affected by solute concentration gradients through membranes, causes changes in the volume of cells, directing the movement or growth of an organism.^25^ Artificial systems and actuators based on osmosis can be energy-efficient and display fast actuation.^26−28^ We note that in osmosis, the osmotic pressure difference is a colligative property; it is dependent on the solute (osmolyte) concentration, not on the type of solute.

**Scheme 1:**
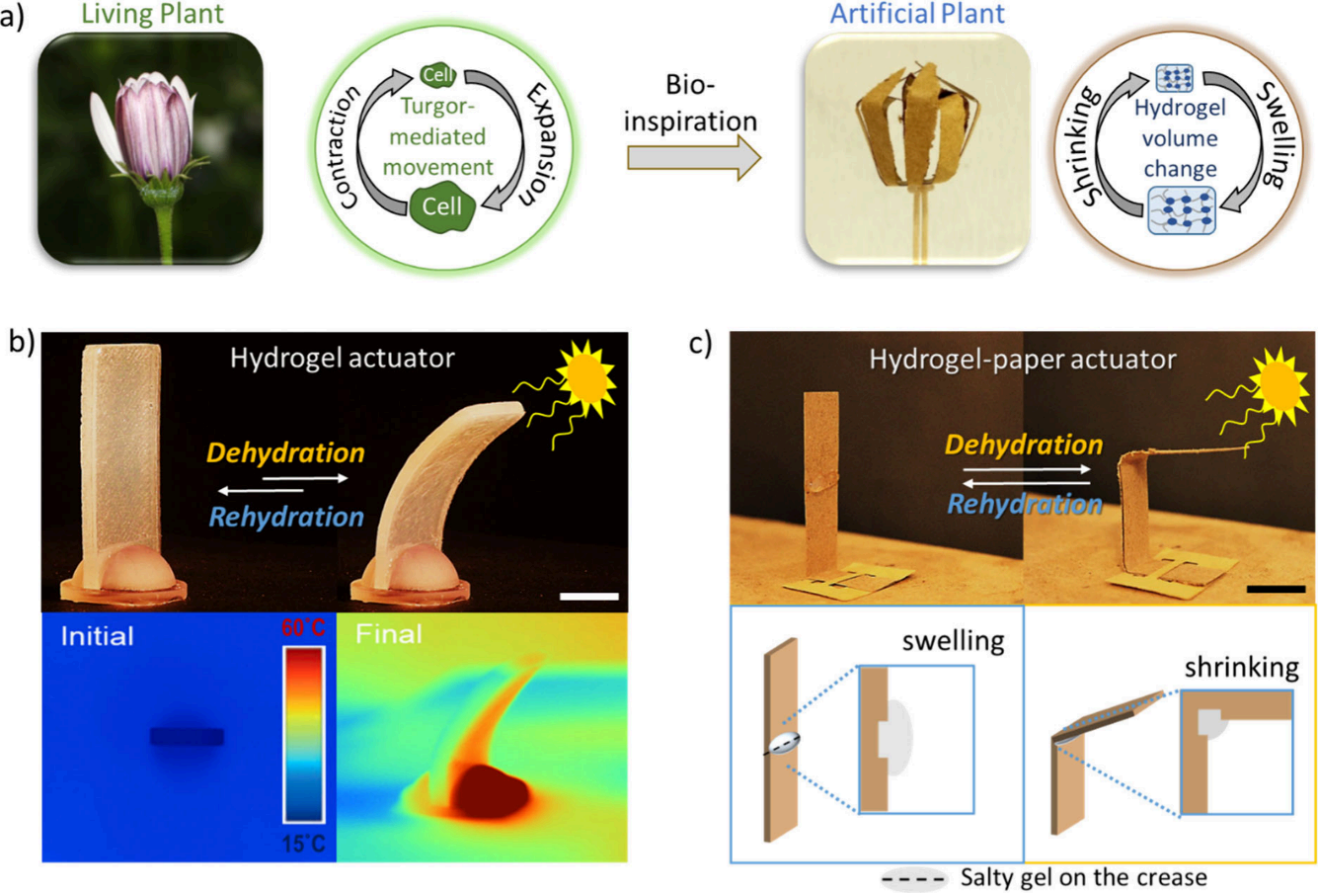
(a) Osmotic Pressure Differences Mediate the Reversible Expansion/Contraction of Plant Cells and Reversible Volume Changes in a Hydrogel Part of a Hydrogel-Paper Artificial Plant by Shrinking/Swelling Mimic the Turgor-Mediated Shape Change in Plants; (b) Adding Salts to Hydrogels Can Be Used to Change the Speed of Dehydration and Rehydration and Overall Bending upon Illumination from One Side of the Hydrogel and Thermal Images of the “Salty” Hydrogel before (from the Top) and after IR Illumination Show Nonhomogeneous Heating and Bending; and (c) “Salty” Hydrogels Cured on Creases of Paper Supports Form the Hydrogel-Paper Actuators The base is a 3D-printed resin and thus has no contribution to the gel action. The speed of bending is affected by many factors, such as the concentration and lyotropic behaviors of the salt ions, and surprisingly (or maybe not, see the Results and Discussion section), it is affected by the diffusion of water in the paper. Scale bar = 1.0 cm.

In our previous reports, we displayed artificial transpiration systems using hydrogels as actuators. The systems displayed autonomous, self-regulating phototropic (following the light source) and nyctinastic (opening and closing upon illumination) motion.^29,30^ The actuation was controlled by light in reversible dehydration–rehydration cycles, affecting the volume change of the hydrogels (Scheme 1a). These systems were made up of just agarose hydrogel or agarose hydrogel on paper. The actuation kinetics of these simple gel actuators were only affected by the geometry of the gel and the incorporation of some additives increasing the IR absorption, e.g., carbon flakes (see Figure S1). Motivated by the high efficiency and speed of the demonstrations of how the solutes in artificial systems can mimic osmosis in biological ones,^26−28^ we decided to include salts in our agarose hydrogel actuators. We hypothesized that salt inclusion should change the speed of transpiration (dehydration and rehydration) and provide a means of tuning the actuation speeds of the motion.

The mechanical actuation of hydrogels can be affected and controlled by various environmental stimuli, such as temperature, light, and pH, as reported in numerous examples in the literature.^31^ For example, thermal phase changes of the polymeric material, such as in poly(*N*-isopropylacrylamide) (PNIPAM) and polyacrylamide (PAM),^32−37^ can be used to obtain elaborate hydrogel actuation. Near-infrared (NIR) light-driven phototropism of thermal-sensitive hydrogels has recently drawn the attention of soft roboticists for its possible applications in energy harvesting, drug delivery, smart light-controlled devices, and artificial muscles.^38−42^ The action displayed by these engineered polymer materials is very sophisticated. The chemical actuation of polymeric soft materials by ions incorporated on the polymer chains as shown by Li et al.^43^ or the salt-incorporated triboelectric generators of nanocellulose composite hydrogels by Wang et al.^44^ proves that salts add additional complexity to the hydrogel action. On the other hand, more common materials, simple and available polymers, and additives are advantageous in soft-robotic applications, where the material amount needed is usually larger than at the lab scale (a few grams). Therefore, our hypothesis to fine-tune the reversible action of agarose with salts, which are also abundant and available to most, seems like a valuable addition to the plant-inspired design and fabrication of soft robots.^45−47^

Another challenging mission for soft material researchers is to create anisotropy in the material to reflect the input by an asymmetrical response. The anisotropy “embedded” in hydrogels can be used for various purposes in soft robotics, such as actuation,^48−50^ sensing,^51,52^ and communication.^53^ Again, numerous examples depict limitless sophistication, as displayed in bimorph soft actuators,^54,55^ in situ copolymerization with 2D materials like MXene nanosheets or photothermal nanotransducers such as Au and Ag,^56−58^ nonhomogeneous hydrogels with different compositions and swelling/shrinkage ratios through origami/kirigami, and localized doping with chemicals.^59,60^ A multitransforming thermosensitive flower by Kim et al.^61^ shows controllable mechanical actuation by introducing an asymmetrically functionalized PNIPAM hydrogel. An octopus-inspired soft swimmer was designed based on an MOF-containing photothermally responsive PNIPAM hydrogel upon NIR irradiation.^62^ Finally, our group recently introduced single material-based anisotropic bending actuators using polydimethylsiloxane (PDMS) sponges.^63^ In this study, we also show that asymmetrical shape-morphing in hydrogels can be attained straightforwardly by the SIE of the in situ loaded salts.

## Materials and Methods

To test the hypothesis that the salt addition can tune the dehydration and the speed of bending in the hydrogel actuators, we prepared two actuator systems: (1) blocks of agarose gel named “hydrogel actuators” and (2) hydrogels cast on the creases of pieces of paper named “hydrogel-paper actuators”, as shown in Scheme 1b,c. In the hydrogel actuator, the side of the gel block illuminated with IR is heated more, and the gel shrinks and bends toward the light (Scheme 1b). The details of the design and production of both systems are displayed below, in the Experimental Section and in Figure S2.

### Choice of Materials

Agarose (ISOLAB, low EEO) was used to prepare the hydrogel actuators. The physically cross-linked 5.0% (w/w) agarose gel was found to be moderately stiff to keep the material in upright and bent metastable positions. Upon illumination with an IR lamp (Tungsram Infrared, 250W) at a constant distance of 30 cm, the dehydration of 40 mm × 15 mm × 3 mm agarose pieces is on the order of minutes to an hour, an acceptable time interval for plant-inspired robotic systems. The other advantage of using agarose is its availability at a low cost in comparison with most other gelating polymers. It is biodegradable and can be handled without any health concerns.^64^ In the second actuator system, commercial cellulose paper (Kraft paper, 200 g/m^2^) was used as the base material due to its high mechanical stability (the interconnected cellulose fibers are displayed in the optical microscope images in Figure S3). Some additives, such as carbonates and aluminosilicates, used to improve paper properties might interfere with ions in the experiments. Therefore, we selected brown paper (see Figure S4 for XRD measurements of the paper used) free of common paper additives, which avoids undesired chemical interactions in the system. We note that qualitatively similar results were also obtained with other types of Kraft paper (papers with additives). We highlight that the hydrogel-paper actuators have a much higher stiffness (with an elastic modulus on the order of GPa)^65^ than the bare hydrogels used in the experiments (Figure S5).

Sodium chloride, sodium thiocyanate, sodium sulfate, potassium chloride, magnesium chloride, and calcium chloride were used as the salts in the experiments. These are some of the representative salting-in, salting-out, and neutral ionic reagents displayed in the typical lyotropic series, as described in the discussions below.

### Preparation of “Salty” Hydrogel Actuators and Hydrogel-Paper Actuators

To prepare the “salty” gels, appropriate amounts of salts to achieve targeted concentrations were dissolved in deionized water (5.0 mL) with 0.25 g of agarose polymer. The mixtures were then heated at 90 °C until all of the solutes were dissolved. The solutions were poured into 3D-printed acrylonitrile butadiene styrene (ABS) molds (internal dimensions of 40 mm × 15 mm × 3 mm), and the molds containing the solutions were kept in a refrigerator (4 °C) for 30 min for complete gelation. The hydrogel actuators were removed from the mold and placed upright on 3D-printed resin stands. To monitor the gel rehydration, the dehydrated gels in the resin stands were placed upright in Petri dishes. The Petri dishes were filled with an appropriate volume of a “reservoir” salt solution (with matching concentrations to the initial salt concentration in the gel) to rehydrate the gels from the bottom ends. For hydrogel-paper actuators, the salty agarose solutions were prepared identically. The solutions were applied via a syringe on the previously formed creases of the Kraft paper and let to gelate in the fridge for 30 min (please see the Experimental Section and the Supporting Information (SI) for preparation details and schemes).

### Hydrogel Actuation, Determination of the Bending Angles, and the Speed of Dehydration

The hydrogel or hydrogel-paper actuators were placed at a constant distance of 30 cm from an IR lamp to affect the asymmetrical dehydration and bending of the actuator. The bending was monitored by a camera placed at a 90° angle to the actuation plane. The photos taken during the actuation period were used to determine the bending speed in terms of the bending angle per actuation time. The speed of actuation was also monitored gravimetrically by the water loss in the hydrogels before and after illumination.

## Results and Discussion

### SIE Behaviors of the Anions and Their Effect on the Dehydration Speed of the Hydrogels

**Figure 1:**
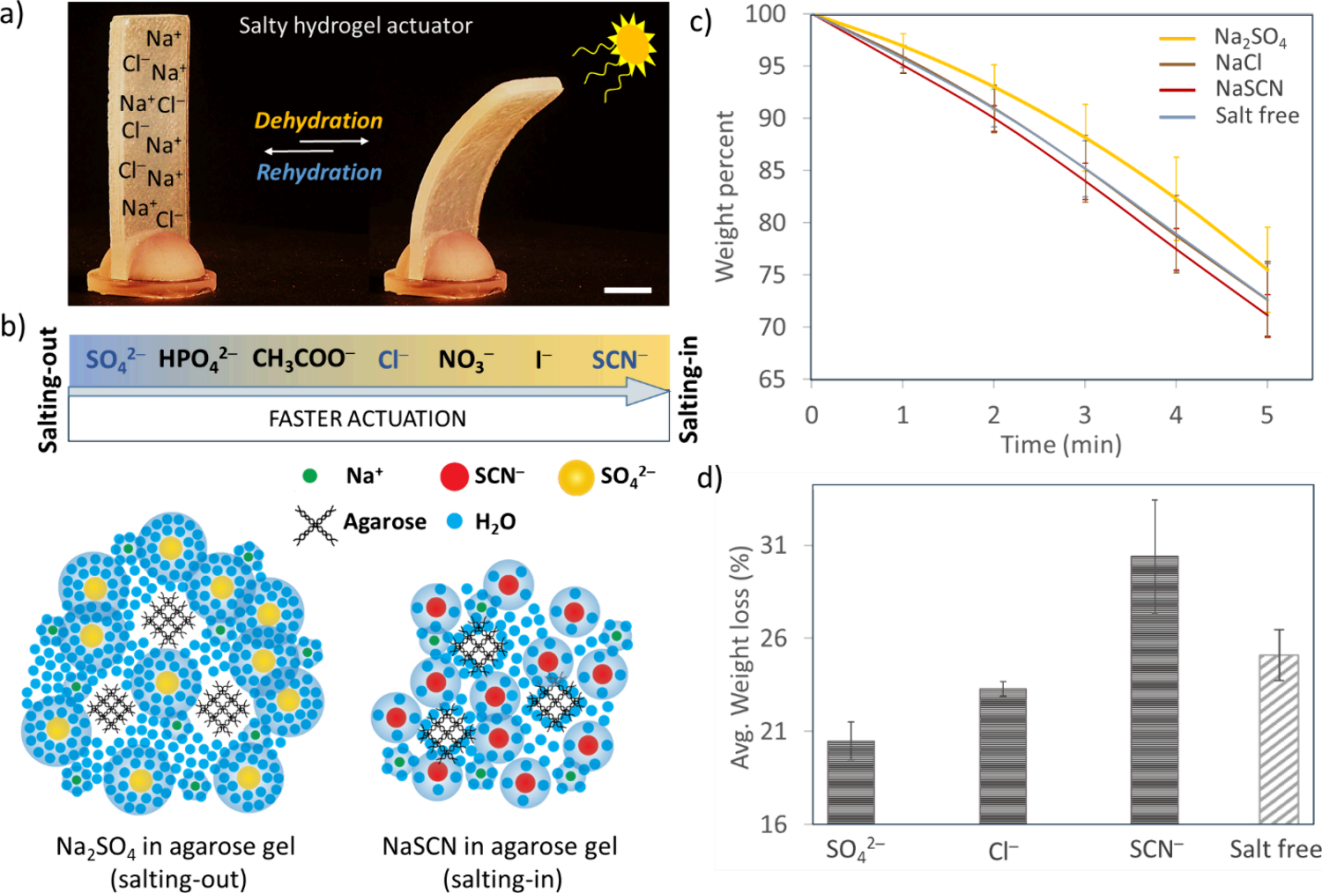
Salts added to an agarose hydrogel affect its dehydration speed. (a) The salty hydrogel actuator’s reversible bending upon dehydration–rehydration. The cycle takes from a few minutes to an hour for salty agarose hydrogels of size 40 mm × 15 mm × 3 mm, depending on the nature of the salt and its concentration. Shown here is the gel with NaCl (0.30 M in gel). Scale bar = 1 cm. (b) An “averaged” SIE series of ions can help to regulate the actuation speed. The forward direction of the arrow indicates the higher salting-in ability of the ions; therefore, faster dehydration and bending can be expected with them. (c) The hypothesis that the added ions could affect the dehydration speed according to the SIE series was preliminarily checked by the TGA analyses of the salty hydrogels. (5.0% agarose, 0.30 M salts). (d) The average weight loss of the salt-added (0.30 M sodium salts of the anions shown) hydrogel actuators (such as that shown in (a)) before and after 30 min of IR illumination. See Materials and Methods and the SI for details of the TGA and weight loss experiments. The error bars represent the standard deviation from at least four identical experiments.

All hydrogel actuators, with or without added salts, bent upon IR illumination from one side, as shown in Figure 1a. Our initial hypothesis that salt addition can affect the hydrogel actuation speed was based on the lyotropic nature of salts. The added ions can preferentially bind water molecules or polymers and change the dehydration speed (Figure 1b). The main hypothesis is that water molecules in the gels containing a weak water hydration for salting-in salt should be readily transpired. On the other hand, adding salting-out ions that are strongly hydrated with water molecules is expected to minimize evaporation and dehydration. As explained above, the SIE series provides a ranking of how the added ions may affect the interactions of water and polymer in a solution and may guide us in selecting the right ions for controlling the direction of change. However, to the best of our knowledge, an SIE series for an agarose polymer is not available in the literature. The existing SIE series for other macromolecules may give a crude understanding of which ions may behave as salting**-**in or salting**-**out.^66,67^ Analyzing the reported SIE series in the literature^15^ and finding that anions give more reliable orders in the series, we first selected one typical salting-in anion (SCN^–^), one typical salting-out anion (SO_4_^2–^), and an anion (Cl^–^) which is almost at the center (neutral SIE behavior) of the series for our experiments. We used sodium salts of these ions since sodium is also at the center of the cationic series. Before going into the detailed actuation analyses with the actuator samples, we made a preliminary thermogravimetric analysis (TGA) check of the gel dehydration speed differences when each of the sodium salts of these ions is incorporated in the agarose (Figure 1c, 5.0% agarose hydrogel, including 0.30 M sodium salts). The TGA plots show that the total weight loss due to evaporating water increases in the case of the ions listed as salting-in in Figure 1b. After 5 min of heating at 55 °C, the average weight loss in the samples of different ions in gels was found to be 28.9% (SCN^–^) > 27.4% (salt-free) > 27.3% (Cl^–^) > 24.5% (SO_4_^2–^). (We note here that the sample size and shape used for typical TGA analysis deviate much from those of the gel samples we used in the experiments. Therefore, the results of TGA can only be used for a trend estimation.)

Encouraged by the TGA results, we next prepared our salty hydrogel actuator blocks (40 mm × 15 mm × 3 mm, 0.30 M sodium salts of the anions in the gel; for details, see Materials and Methods). We determined the weight loss of our salty hydrogel actuators by weighing the gel blocks before and after illumination for 30 min by an IR lamp placed directly toward the gels at a distance of 30 cm. The weight loss order was found as SCN^–^ > salt-free ≈ Cl^–^ > SO_4_^2–^ (Figure 1d).

### SIEs of the Anions and Their Effect on Actuation Speeds of Hydrogel Actuators

**Figure 2:**
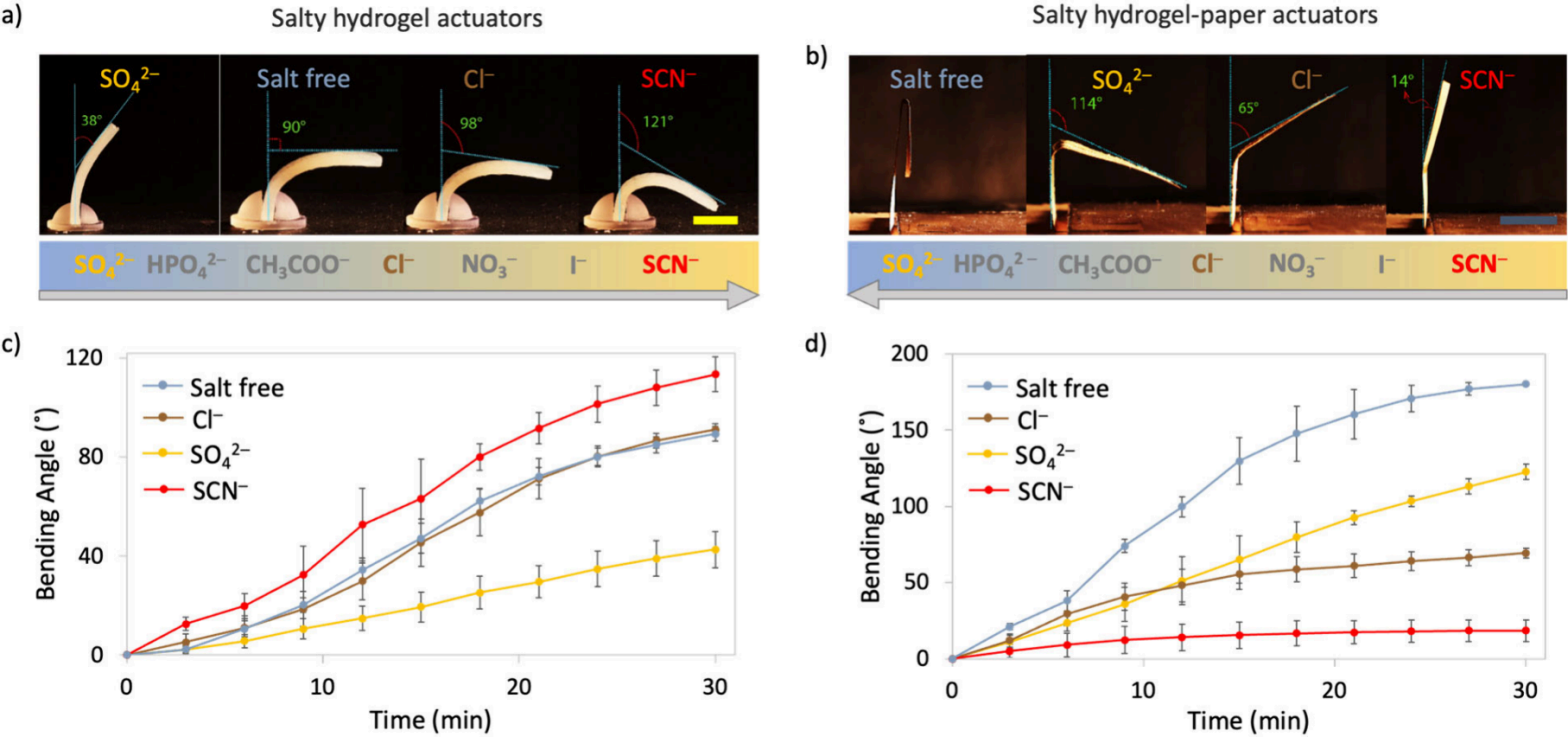
Bending behavior of salty hydrogel and hydrogel-paper actuators containing sodium salts of SCN^–^, Cl^–^, and SO_4_^2–^ after illumination with an IR lamp for 30 min (5.0% agarose hydrogel, 0.30 M sodium salts). (a) The hydrogel actuators show an actuation speed trend in accordance with the SIE series (SCN^–^ as the salting-in and SO_4_^2–^ as the salting-out ion). (b) The hydrogel-paper actuators, on the other hand, show a dehydration speed trend opposite to the expectations built on the SIE series. Scale bar = 1 cm. (c, d) The actuation speeds (bending angle/time) of the hydrogel and hydrogel-paper actuators are significantly different for different ions in the actuators. The error bars represent the standard deviation from at least five experiments with identical conditions (5.0% agarose hydrogel including 0.30 M sodium salts on Kraft paper).

Since it is impossible to analyze the effect of cations and anions simultaneously, we started with the assessment of the effect of anion addition. The actuation (or bending) speed was determined as the bending angle per minute for the actuators. When subjected to IR illumination, the hydrogel actuators containing sodium salts of SCN^–^ bent faster than those with SO_4_^2–^, as shown in Figure 2a. This is in accordance with the SIE series that the salting**-**out SO_4_^2–^ ions interact with hydrating water molecules stronger than the salting**-**in SCN^–^ ions.^15^ Cl^–^-containing hydrogels bent with speeds higher than those of SCN^–^ and lower than those of SO_4_^2–^, which agrees with the central place of chloride ions in the general SIE series.

The results of similar experiments with salty hydrogel-paper actuators, on the other hand, were quite surprising. When these actuators were subjected to illumination, the actuation speeds of actuators were in the order SO_4_^2–^ > Cl^–^ > SCN^–^ (Figure 2b). This trend was opposite to that observed with the hydrogel actuators (Figure 2a) and that which can be expected from the SIE order of the ions. Another interesting result was that the fastest actuation was observed for the actuator without any salt (Figure 2). The speed of actuation did not change significantly over the whole duration of actuation for the hydrogel actuators (Figure 2c), whereas the salty hydrogel-paper actuators show an initial rapid actuation, followed by a decrease in speed (Figure 2d). We note that for both hydrogel and hydrogel-paper actuators, the SIEs for dehydration speeds become more pronounced in the directions that they are observed (faster or slower speeds) as the concentration of the ions increases in the range 0.10 to 0.30 M (Figure S6a). The weight loss measurements also suggest higher dehydration at higher concentrations (Figure S7). When the gels contain more than 0.30 or less than 0.10 M, the effect almost levels out and becomes insignificant. The SIE was also observed similarly in the experiments where the ionic strengths rather than the concentrations of the ions were kept constant (Figure S6b).

**Figure 3:**
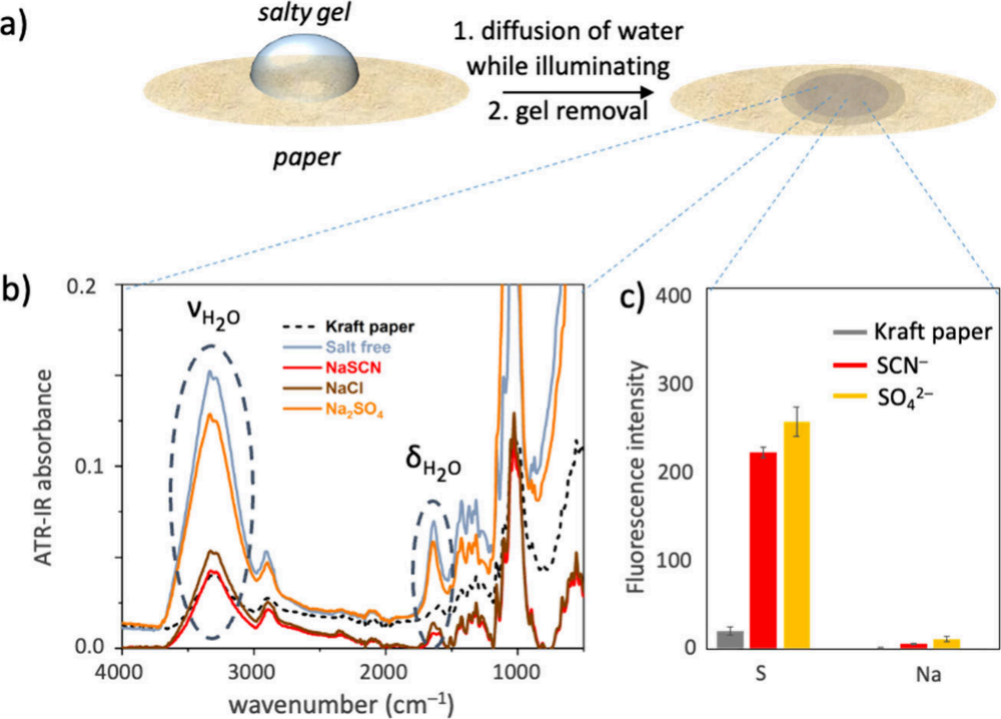
(a) The setup for analysis of water transportation from the hydrogel into the paper in hydrogel-paper actuators. The salty solutions (Na_2_SO_4_, NaCl, and NaSCN, 0.30 M in 5.0% gel) were allowed to diffuse for 30 min into the paper before the gels were removed. (b) ATR-FTIR spectra of the paper/gel interface right after removal of the gels. Higher intensities of water stretching and bending vibrations on paper for the Na_2_SO_4_-added gel account for the higher transportation of water into the paper with this gel. (c) Average fluorescence intensity of sulfur in XRF analysis of the paper/gel interface after removal of the gels shows that SO_4_^2–^ and SCN^–^ diffused approximately in the same amount into the paper.

The trend observed for the hydrogel-paper actuators’ dehydration speeds, which was opposite to what can be expected from the ions’ places in the SIE series, was quite intriguing. The results show that the paper involved in these actuators not only behaves as a mechanical support but is also involved in ion–water–polymer interactions, affecting the evaporation speeds. In hydrogel actuators, water loss is due only to the evaporation facilitated by IR illumination. In hydrogel-paper actuators, water can also diffuse from the hydrogel into the paper. (In the absence of illumination, this diffusion can lead to bending of about 45° in 24 h.) Like the dehydration speed upon illumination, the water transportation speed is controlled by the chemistry of the ions in the hydrogel. To assess the water transportation speed differences for different salty hydrogels, we cast 50 μL of the salty agarose pregel solution on the Kraft paper (Figure 3a). After 30 min, we removed the gels from the paper and analyzed the paper interface, where the hydrogels were placed by X-ray fluorescence (XRF) and attenuated total reflectance-Fourier transform infrared (ATR-FTIR) spectroscopy (Figure 3c and Figure S8). The XRF analysis displayed the S content in the region of the paper in which the water diffuses into the hydrogel. The analysis showed that SCN^–^ and SO_4_^2–^ were approximately the same amount at approximately the same diffusion depth (see Figure S9 and the Supporting Information for a detailed discussion of the XRF results). On the other hand, the intensities of water stretching and bending vibrations in the ATR-FTIR spectra of the interface displayed in Figure 3b showed that when sulfate ions diffuse into the paper, a significant amount of water (stretch: 3000–3700 cm^–1^ and bend: 1640 cm^–1^) is transported with them. In contrast, when thiocyanate ions diffuse into the paper, they are not accompanied by much water. Thus, it can be stated that water brought by sulfate ions can be efficiently drained out of the gel, leading to fast bending, and a lesser amount of water is brought by SCN^–^ ions, which yields slower bending of the actuators. Following this, one can expect the salt-free gel to have the largest diffusion speed of water into the paper since the hydrating water of any ions is not present. This should result in the fastest bending speed, which was the exact experimental result we obtained (Figure 2d).

### SIEs of Anions and Their Effect on the Mechanical Properties of the Hydrogel Actuators

Other factors can affect the actuation speed in hydrogel actuators in addition to the SIE. For example, the mechanical properties of the gel can be altered with the addition of salt to the gel. To probe this possibility, the stress–strain plots of the hydrogels using a dynamic mechanical analyzer (DMA) instrument at 55 °C (to resemble the temperature at which the bending took place) were obtained (see the Experimental Section and Figure S10a for further details of the mechanical tests). The Young’s modulus (*E*) values show that the SCN^–^-added gel had the lowest *E* (3.9 kPa), while the SO_4_^2–^-doped gel had the highest *E* (9.1 kPa) among all. Next, three-point bending tests were also performed at 55 °C for each gel (Figure S10b). The bending angle and the bend allowance were determined from the test results. (The bend allowance describes the radius of the circular segment, which the flat sample curls into after the bending.)^68^ The gel with thiocyanate ions showed the highest average bending angle after the test, 94° (SCN^–^) > 58° (Cl^–^) > 50° (SO_4_^2–^); the lowest inner radius, 1.25 cm (SCN^–^) < 1.75 cm (Cl^–^) < 1.95 cm (SO_4_^2–^); and the lowest bend allowance, 1.98 cm (SCN^–^) < 2.68 cm (Cl^–^) < 3.22 cm (SO_4_^2–^). Lastly, storage (*G*′) and loss moduli (*G*″) were also determined as a function of frequency for the salt-doped agarose gels (Figure S11). The storage modulus of SCN^–^-doped gels was significantly lower than that of the other gels, while the gels with SO_4_^2–^ had the highest *G*′ (Figure S11a). The results indicate that the gel with salting-out ions is stiffer and more resistant to bend in response to external forces. On the other hand, salting-in-doped gels are more flexible and can undergo greater elastic deformation under the same stimuli. Interestingly, the loss modulus of SCN^–^-doped gels is also lower than the rest, showing a less viscous gel and less energy dissipation compared to the other gels (Figure S11b). This is in contrast to what is expected from the phototropic experiments. However, the phototropic bending of gels results from differential expansion, where one side of the gel expands more than the other side due to light absorption and heating. The ability of the gel to respond to such an expansion elastically is controlled by *G*′. In other words, *G*′ dominates *G*″.

The mechanical test results harmonize with each other and with our bending experiments of the hydrogel actuators: the gels doped with thiocyanate ions that bend more have the least stiffness, which means that they need a lower force to deform. On the other hand, SO_4_^2–^-doped gels have the highest stiffness, and higher force is required to deform them. It can be concluded that the SIE affects the mechanical properties, in addition to the evaporation speed, and may contribute to the actuation speed differences observed with different anions in the gels.

### SIEs of Cations and Their Effect on Actuation Speeds of Hydrogel Actuators

**Figure 4:**
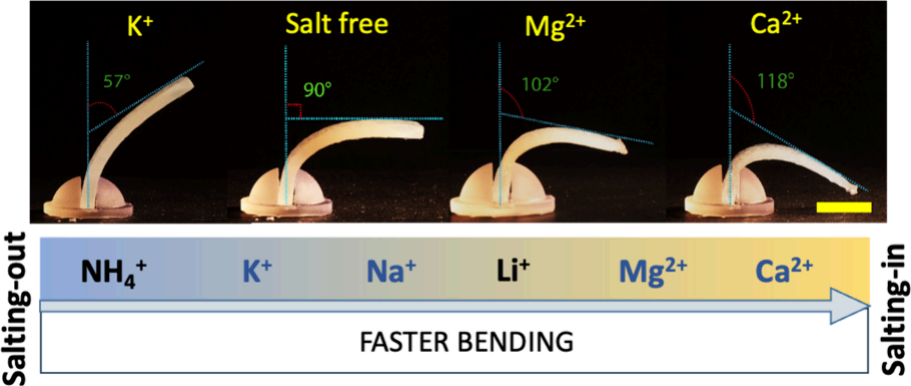
Bending of salty hydrogel containing chloride salts of K^+^, Mg^2+^, and Ca^2+^ after illumination with an IR lamp for 30 min (5.0% agarose hydrogel, 0.30 M chloride salts). The hydrogel actuators show an actuation speed trend in accordance with the SIE series (K^+^ as the salting-out ions and Mg^2+^ and Ca^2+^ as the salting-in ions). The salt-free actuator is placed as a control experiment.

Similar to the approach we followed for the anions, we examined the reported SIE series in the literature^15^ for the cations to determine the cations that are suitable for making a difference in actuation speeds. It is reported that the cations’ SIE behavior is more complicated and less significant compared to that of the anions. Nevertheless, we selected two typical “salting-in” cations (Ca^2+^, Mg^2+^) and two typical “salting-out” monovalent cations (K^+^), in which Na^+^ is almost in the middle of the SIE series (Figure 4).^15^ Interestingly, the “salting-out” cations are reported as the weakly hydrated ones in many studies.^11,65,69,70^ We used the chloride salts of these ions since chloride is also at the center (neutral SIE behavior) of the anionic series. The hypothesis was tested again for the cations this time. The added ions, which are salting-out, should slow the actuation and vice versa. Thermogravimetric analysis (TGA) showed that when each of the chloride salts of the mentioned ions were incorporated in the agarose, the nature of the cation did not affect the dehydration speeds as much as it did for the anions (Figure S12, 5.0% agarose hydrogel, including 0.30 M chloride salts). After 5 min of heating at 55 °C, the average weight losses in the samples of different ions in gels were found to be similar for all salts, without a definite trend and with large standard deviations in the measurements. Still, the actuation speed (the bending angle vs time) of the salty gel actuators followed the overall SIE trend as expected (Figure 4). Similar to the anions, the effect of the SIE behavior on the actuation differences becomes less significant below 0.30 M (Figure S13). The hydrogel-paper actuators of the cations did not provide statistically meaningful results, presumably because of the more complex SIE behavior of the cations when another polymer (cellulose of the paper) was present.

### Reversible Actuation of the Salty Hydrogel Actuators

Reversible and continuous action are primary requirements for soft-robotic actuators. Since this study aims to study the effect of the ions’ SIE series on hydrogel actuation and covers only the experiments with physically cross-linked agarose hydrogels, plastic deformations are inevitable after many actuation cycles. For all the salty actuators in this study, the actuation was reversible for only up to a few cycles (Movie S1, Figure S14). The actuators necessitate more optimization to be more elastic for use, e.g., as a soft-robot part. We should also note that the gels shall never be allowed to dry completely if reversibility is desired. Therefore, we let the gels dehydrate for 20 min (instead of 30 min for other experiments) and rehydrated for 60 min. In the case of salting-out*-*doped gels, the formation of salt crystals as a result of precipitating out the polymer prevents gels from performing reversible actuation after the third cycle (Movie S1). Also, for this reason, we had to decrease the dehydration and rehydration times for each subsequent cycle.

### Anisotropic Actuation of the Salty Hydrogel and Hydrogel-Paper Actuator Systems

**Figure 5:**
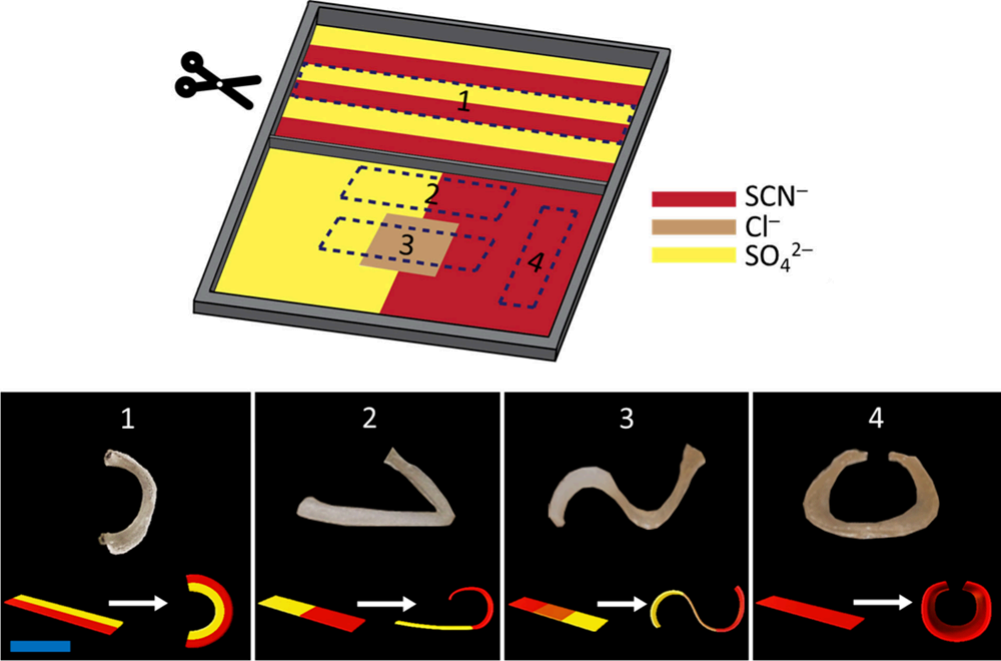
Anisotropic shape-morphing salty hydrogel actuators: four different 3D shapes were obtained through different actuation speeds of the different parts of the same initial shape samples. Initial ion concentration was 0.30 M in each salty domain. Scale bar = 1 cm. See the SI for the preparation details of the anisotropic hydrogel samples.

Hydrogel actuators can be prepared to contain different salts in the neighboring regions. Such a design creates a geometrical dehydration anisotropy in the sample. As examples, we prepared four samples of identical size and shape (40 mm × 7.5 mm × 3 mm) but with different salt solutions at the different regions of the gel, as shown in Figure 5 (see the SI for the preparation details of the anisotropic hydrogel samples). The regions of the different ions were not separated physically or with a membrane to keep the design simple. (Since there was no physical membrane separating gels with different moieties in this design, the ions were free to diffuse from their gel region to the neighboring one. We performed a Raman mapping of ion diffusion across the gel that was doped with thiocyanate ions on one half and with sulfate ions on the other (Figure S15). The data imply that about 15–20% of the ions diffused to only 2 mm of the opposite side of the interface after 30 min of light illumination. This means that the ions diffused were not of significant amount to affect the results for the duration of actuation.) When the samples were illuminated by an IR lamp (for 30 min) at the same distance, the actuation resulted in four different 3D shapes, as expected. These simple demos showed that many different shapes are accessible starting from the same initial shape using a single input or that symmetry of the regular shapes can be broken when the SIE behavior of the ions was used to create the required anisotropy.

**Figure 6:**
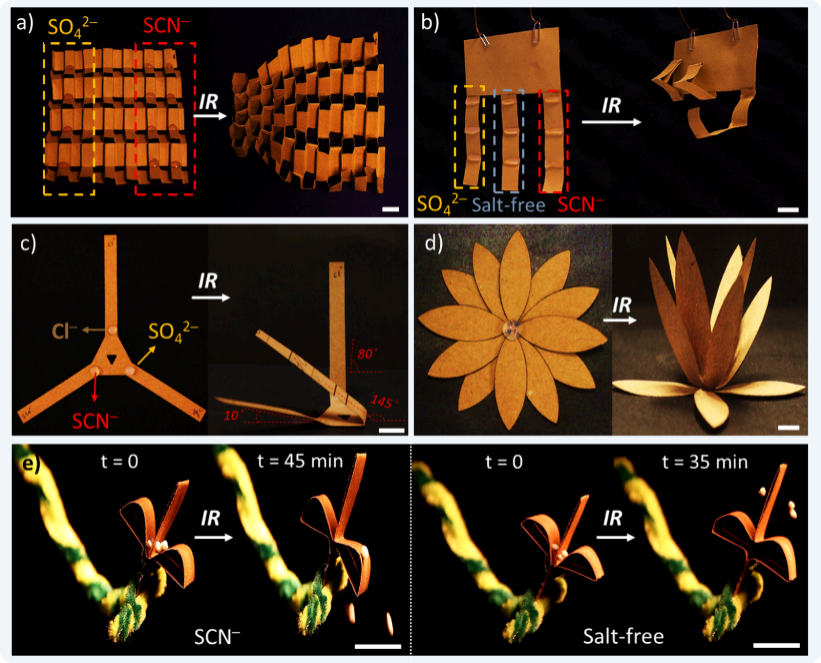
Anisotropic/unsymmetrical/sequential actuation of some salty hydrogel-paper actuator systems. SIE behaviors of the ions in the hydrogels located in the creases of the paper affect the anisotropy of the final structure upon heating in an oven or IR illumination. (a) Kirigami structure with SO_4_^2–^- and SCN^–^-doped gels cast on creases on the two left and right rows of the paper design. The illumination caused the side with SO_4_^2–^ to dehydrate faster and shrink more (see Figure S16 for the design and preparation details). (b) A gripper, in which the “fingers” having different salty gels at the creases fold at different speeds upon heating. (c) A three-arm design displays dynamic anisotropic folding upon illumination. (d) A three-layered lotus-shaped design, where different salty gels (SCN^–^, Cl^–^, and SO_4_^2–^ from bottom to top) were cast at the center of each layer. The lotus layers “close” sequentially upon illumination. (e) Artificial seed-shooting plant mimicking “ballochory” in plants. The gels were cast on the bend arc of the paper attached to the stem; dehydration caused the detachment and the release of the seeds, as shown, at different times for salty and salt-free gel (see also Movie S2). Scale bar = 1 cm.

Similarly, the concept of SIE-induced anisotropic actuation can be expanded to hydrogel-paper actuator systems of kirigami-designed Kraft paper (Figure 6 and Figure S16). Some of the samples displayed in the figure were actuated by keeping them at 60 °C in an oven for 1 h to provide a homogeneous heat distribution for the large samples. Here, we chose to make a few different designs. The first design was created through kirigami. SO_4_^2–^ and SCN^–^ ion-containing gels were placed at the creases of the paper on the left and right sides, respectively (Figure 6a). The side of the paper that has gels doped with SO_4_^2–^ folds and shrinks more upon heating, demonstrating an anisotropic bending and unsymmetrical shape change upon a single input. Figure 6b displays a gripper with three “fingers” having different salty gels. The fingers folded at different speeds upon heating. The differences in folding speeds in a three-arm design (Figure 6c) showed dynamic asymmetric bending in the system upon illumination. A three-layered lotus-shaped design, shown in Figure 6d, had different salty gels cast at each layer so that a sequential closing of the lotus could be programmed. Finally, in Figure 6e, we tried to demonstrate artificial seed-shooting, mimicking “ballochory” in plants. The gels were cast on the bend arc of the paper attached to the stem in this plant; the different dehydration speeds of the salty and salt-free gels caused different times of detachment and the release of the seeds in the corresponding systems.

## Conclusion

The specific ion effect (SIE) is a phenomenon that has been known for more than a century. It is a vital component of many biochemical processes and has been used to develop industrial applications. Yet, looking at the “bottom” of the SIE, the chemistry is quite complicated. Here, we studied the possibilities of using the SIE of some ions in hydrogels to control the hydrogel dehydration speeds under illumination, which showed that such an SIE-controlled regulation is possible. This regulation also contributes to the change in mechanical properties of the gel, also mediated by SIE. With the inclusion of a paper support, other polymer–ion interactions and differences in diffusion speeds become major factors affecting, even reversing, the SIE-expected actuation order. Finally, we showed that the SIE regulation of dehydration and actuation could be used in combination with the geometrical design of both hydrogel and hydrogel-paper actuator systems, leading to higher levels of complexity and asymmetry.

Using a well-known physicochemical phenomenon and basic materials that all can find, we showed straightforward access to the complex behavior of soft materials and the diversification of hydrogel behavior needed in hydrogel robotics. On the other hand, reversibility and durability, also imperative in soft-robotic applications, are limited in these systems due to the physical cross-linking of the agarose gels. It is hard to compare the actuation rates of the gel actuators manufactured in this study with those of thermal-sensitive hydrogels or thermal phase change polymeric materials^32−42^ since the geometry of the actuator majorly affects the actuation speed, and the examples have different geometries. The actuation speeds of agarose hydrogels can be augmented with other heat-absorbing additives such as graphite flakes. Nevertheless, the simple undoped agarose platform offered easy preparation and low-cost materials needed for diligent testing of the SIE in hydrogel actuators, which was the target of this work. We believe our study can be useful in further scientific inspections of SIE, lightweight, bioinspired hydrogel system designs, and as a conceptual minimalistic mark in soft robotics.

## Experimental Section

### Chemicals and Reagents

Agarose (ISOLAB, low EEO), sodium chloride (Scharlau, extra pure), sodium thiocyanate (Thermo Scientific, 98% purity), sodium sulfate anhydrous (Merck), potassium chloride (PanReac AppliChem ITW Reagents), magnesium chloride hexahydrate (CARLO ERBA Reagents), calcium chloride dihydrate (Sigma-Aldrich, 99% purity), and Kraft paper (200 g/m^2^). All the hydrogels and solutions were prepared using deionized water.

### Hydrogel Preparation

An appropriate amount of salt was dissolved in 5.0 mL of deionized water. Then, 0.25 g of agarose polymer (to make 5% gel) was added to the solution and heated at 90 °C until all the polymers were dissolved. Subsequently, the solution was poured into a 3D-printed mold, designed based on the desired geometry, and kept in a fridge for 30 min to gel.

### Determination of Bending Angle

A Canon camera was fixed at a zero angle with a proper distance from the actuator sample and captured an image every 3 min for 30 min. The photos were then transferred to a computer, and the angles were calculated by using Adobe Illustrator software.

### ATR-FTIR Analysis

A 50 μL portion of a pregel solution (0.30 M salt and 5.0% agarose) was cast on dry Kraft paper. After gelation, the sample was placed in front of an IR lamp at a 30 cm distance for 30 min. The gel was then removed from the paper, and the gel–paper contact was analyzed using a Bruker ALPHA II FTIR spectrometer with an ATR accessory.

### XRF Analysis

The same sample preparation as the ATR-FTIR analysis with 20 mm diameter paper discs was carried out for the XRF analysis (0.30 M salt and 5.0% agarose). The analysis was performed with a Rigaku ZSX Primus II XRF instrument.

### TGA

Hydrogels with and without salts (0.30 M salt and 5.0% agarose) were prepared in Petri dishes. Then, 8 mg samples were cut with a blade out of the gels in the dishes. The analysis was performed using a TA Instruments TGA Q500 instrument on a platinum pan, heating for 5 min at 55 °C.

### DMA Analysis

The tensile and bending analyses were done on a TA Instruments DMA Q800 instrument at the stabilized temperature of 55 °C.

#### Tensile Test

The strain induced was measured as a function of the tensile force with a 1 N/min force rate. Rectangular sheets of 40 mm × 15 mm × 3 mm were cut out of freshly prepared hydrogel bars (0.3 M salt and 5% agarose).

#### Three-Point Bending Test

A force rate of 1 N/min was performed on rectangular sheets of 35 mm × 15 mm × 3 mm cut out of freshly prepared hydrogel bars (0.3 M salt and 5% agarose).

## Supporting Information Available

Optical microscopy images and XRD patterns of Kraft papers; TGA, DMA, XRF, ATR-FTIR, and Raman spectroscopy data of samples; the effect of concentration of the salts on the bending; average weight loss; comparison of bending of salt-free hydrogel and salt-free hydrogel-paper actuators; average bending angle and weight loss of chloride salts; reversibility plots of the salty hydrogel actuators; and the design of kirigami-based hydrogel-paper actuators (PDF)
Movie S1: a video of the self-regulation of the hydrogel actuator (MP4)
Movie S2: a video of the seed-shooting plant robot (MP4)
