# Supplementary material for: Lyotropic “Salty” Tuning for Straightforward Diversification and Anisotropy in Hydrogel Actuators
Source: Langmuir. 2025 Jan 1;41(1):162–71. doi: 10.1021/acs.langmuir.4c03291 (PMC11736847; doi:10.1021/acs.langmuir.4c03291)
Supplement: Supplementary file 1 — la4c03291_si_001.pdf [file la4c03291_si_001.pdf]

Supporting Information

Lyotropic ‘salty’ tuning for a straightforward diversification and anisotropy in hydrogel actuators

Pedram Tootoonchian,[a] *Levent Bahçeci,*[a] *Andriy Budnyk,*[b] *Halil I. Okur,** [a,b]

*and Bilge Baytekin** [a,b].

[a] Chemistry Department, Bilkent University, Ankara 06800, Turkey

[b] UNAM – National Nanotechnology Research Center, Bilkent University, Ankara, 06800, Turkey

Number of pages: 17 (page S1 to page S17)

Number of Figures: 16 (Figure S1 to Figure S16)

Number of Movies in the Supporting Information: 2 (Movie S1 and S2)

Table of contents

1. Preparation of a hydrogel-paper actuator

2. Surface morphology of Kraft paper samples

3. A structural inquiry into the composition of Kraft paper samples

4. Mechanical properties of the paper versus gel (Young's modulus)

5. The effect of the concentration of the salt on the bending

6. The effect of the concentration of the salt on the weight loss

7. Mechanical properties of salt-doped gels (Young's modulus)

8. Anisotropic actuation of the 'salty' hydrogel actuators

References

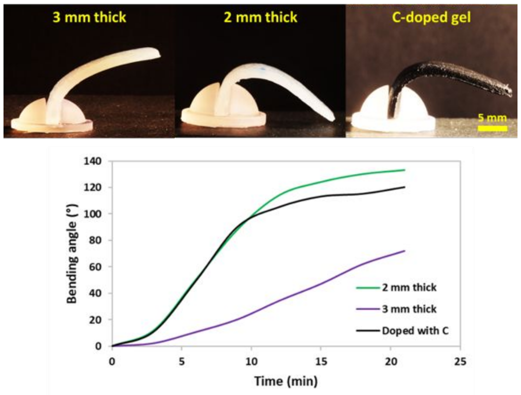

Figure S1. The actuation kinetics of a 2.0 mm thick gel, a 3.0 mm thick gel, and a gel with added graphite flakes (Asbury Chemicals, acid washed graphite flakes, 1:1 w/w flakes: agarose gel) The images on top represent the bending of gels after 20 mins.

1. Preparation of a hydrogel-paper actuator

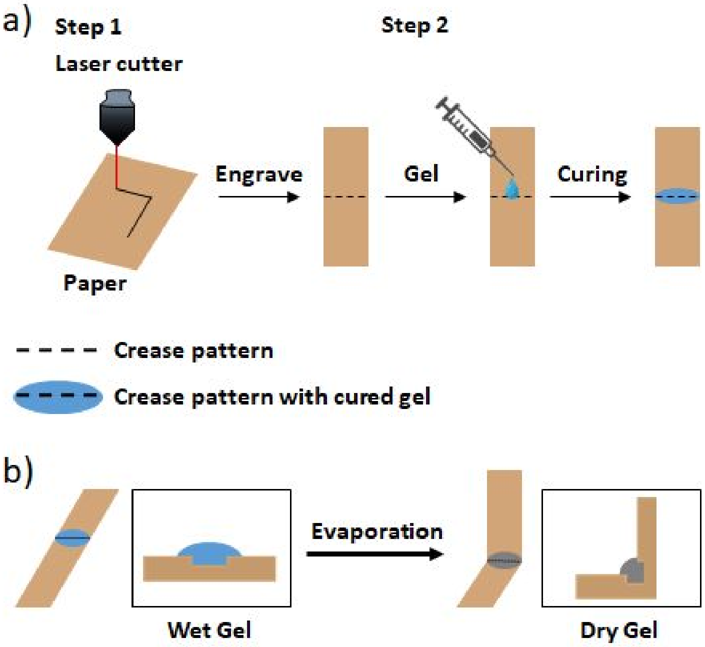

Figure S2. Preparation of the hydrogel-paper actuator. (a) Schematic representation of step-by-step preparation of the folding paper structures. On a piece of paper (200 g/m^2, 10 mm wide, 60

mm long, and 0.5 mm thick), a folding pattern is created by engraving the paper at desired folding lines (here shown, a perpendicular graving made on the halfway of the long axis) with a laser cutter (Step 1). Then the gel (agarose, 100 μL, 5%) is drop cast and let to cure on the engraved creased pattern (Step 2). (b) Schematic representation of the folding mechanism of the hydrogel-paper actuator. When the gel is hydrated (swollen), the actuator stays flat. However, when the gel is dehydrated (contracted), the actuator starts bending.

2. Surface morphology of Kraft paper samples

Photographs of Kraft paper samples were captured by a Canon PowerShot A640 camera mounted on a Zeiss Axio Imager A1m optical microscope.

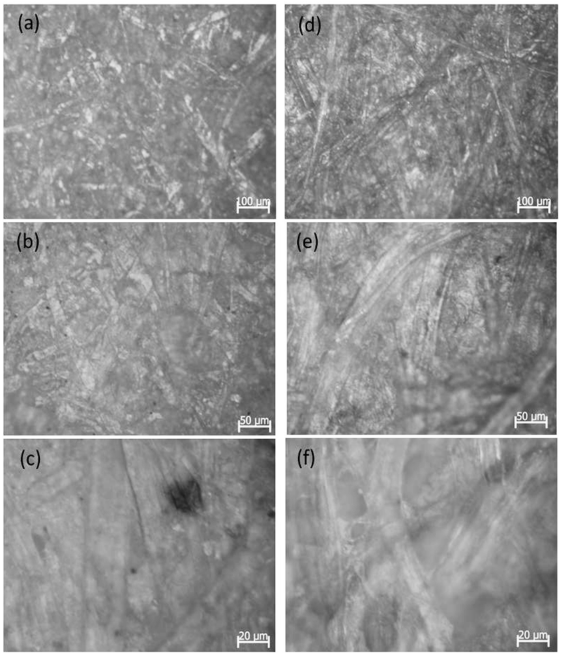

Figure S3. Optical microscopy images of the green-yellow Kraft paper sample I (a-c) and the dark-brown paper II (d-f) taken with increasing magnification (from up to down 10x, 20x, and 50x).

3. A structural inquiry into the composition of Kraft paper samples

Kraft paper is known to be composed of cellulose fibers with some mineral additives to improve the mechanical and aesthetical properties of the product. The XRD measurements of two Kraft paper samples (I and II) were performed on an X'PertPRO (PANalytical) instrument. For the sake of comparison, the microcrystalline cellulose powder was measured, too. The XRD patterns of the most common clay mineral additives[1] as calcite, kaolinite, and muscovite were retrieved from the online mineralogy database of the RRUFF™ Project (https://rruff.info). The measured and the reference XRD patterns are reported vertically shifted for clarity.

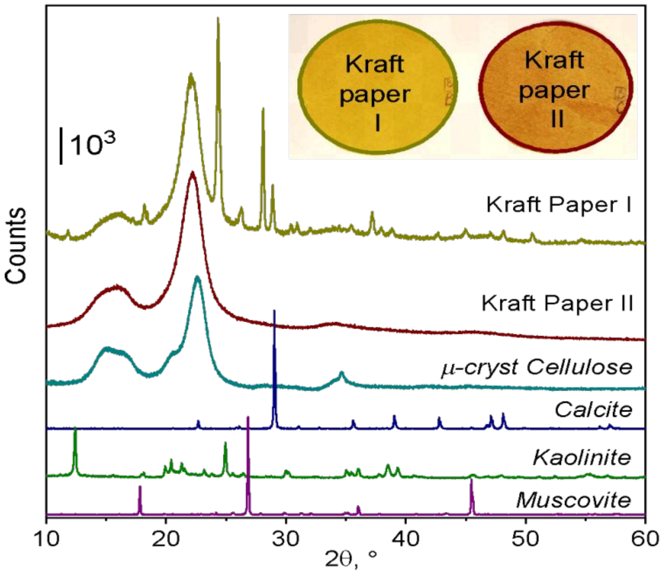

Figure S4. XRD patterns of two Kraft paper samples (I and II) and references: cellulose, calcite, kaolinite, and muscovite. Photographs of Kraft papers are shown in the inset.

The XRD patterns of both Kraft paper samples are dominated by broad peaks of cellulose. Instead, they are distinguished by the presence of mineral additives in the Kraft paper I sample and their absence in Kraft paper II. To avoid any interference from mineral additives on the ion transfer, Kraft paper II has been selected as the support in this study.

4. Mechanical properties of the paper versus gel (Young's modulus)

The tensile test for the Kraft paper and the salt-free gel was performed at room temperature.

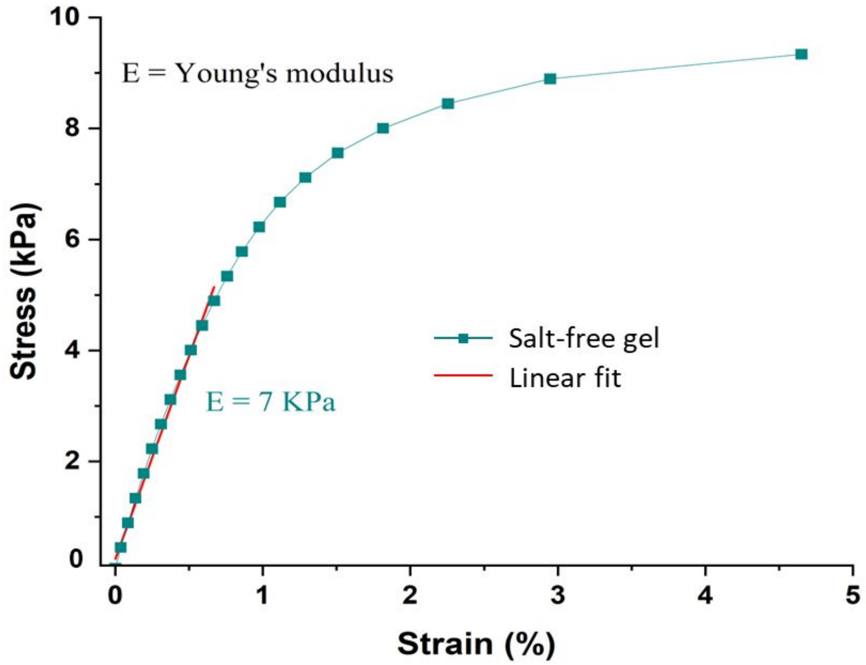

Figure S5. Stress-strain plots for the hydrogel material (5% agarose) used in the hydrogel actuators

5. The effect of the concentration of the salt on the bending

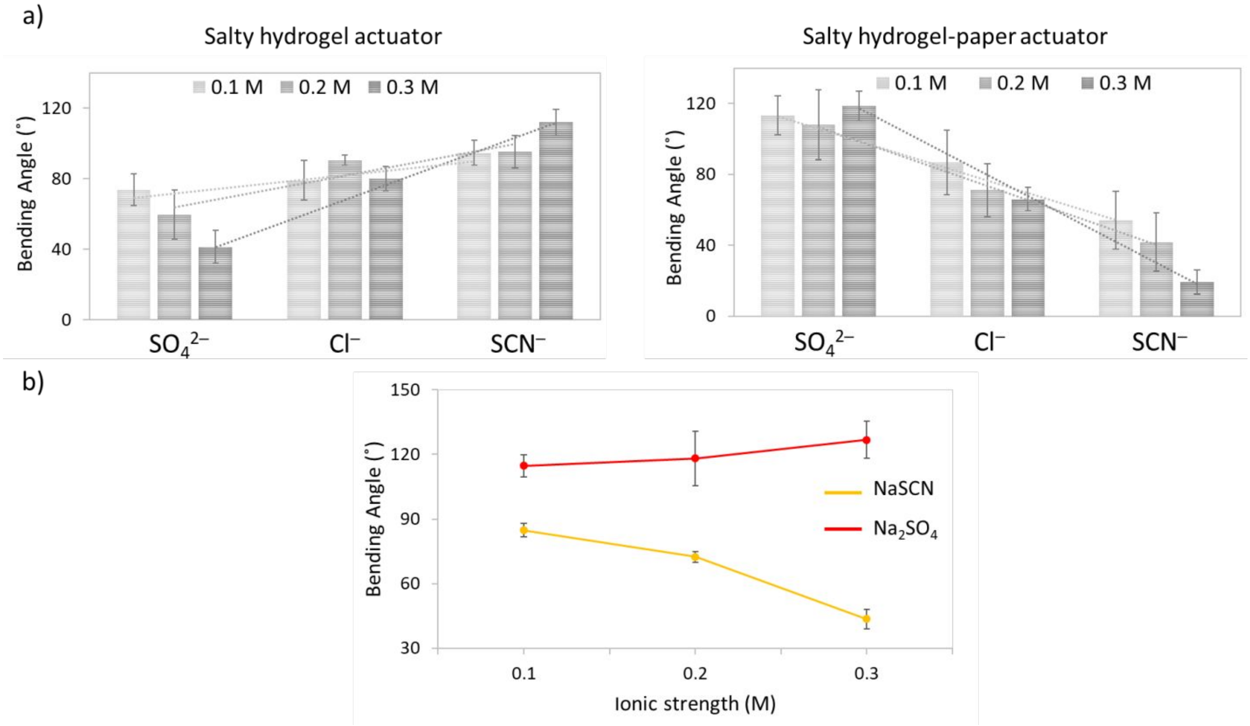

Figure S6. a) SIE is more effective when the ion concentrations are increased from 0.10 M to 0.30 M for ions at the opposite ends of the series, SO_4^{2-} and SCN^-, in both types of actuators. The error bars represent the standard deviation from at least four identical experiments. b) The SIE was also observed similarly in the experiments where the ionic strengths rather than the concentrations of the ions were kept constant.

6. The effect of the concentration of the salt on the weight loss

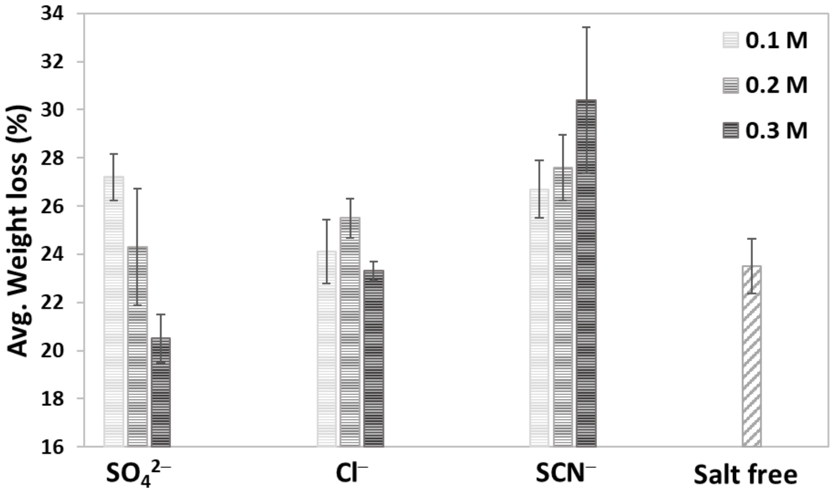

Figure S7. Increasing the concentration of ions from 0.10 M to 0.30 M results in higher weight loss of the gels before and after 30 min of light illumination. The error bars represent the standard deviation from at least four identical experiments.

Study of the ionic transfer from the gel into a paper support

When a hydrogel contacts a paper support, water will diffuse into the paper, accompanied by the ions. We employed both X-ray Fluorescence (XRF) and infrared (FTIR) spectroscopy to identify the population of ions in the paper and respective functional groups of a Kraft paper after its contact with the hydrogel.

The fresh hydrogels were prepared for each measurement. The 20 mm discs were cut from a Kraft paper sheet with a laser cutter. The 'blank sample' presented the initial content of elements in the paper. The samples immersed in HCl acid to remove the calcium inclusions and then dried were called 'acid-washed samples.' A gel solution was let to gelate on the blank and the acid-washed

samples. After gelation, the gel was heated by an IR lamp for 10 min, and then it was removed from the paper. The contact surface was analyzed by XRF and FTIR-ATR.

Infrared Spectroscopy (FTIR-ATR). For IR spectra, a Bruker ALPHA II FTIR spectrometer with an Attenuated Total Reflectance (ATR) accessory was used.

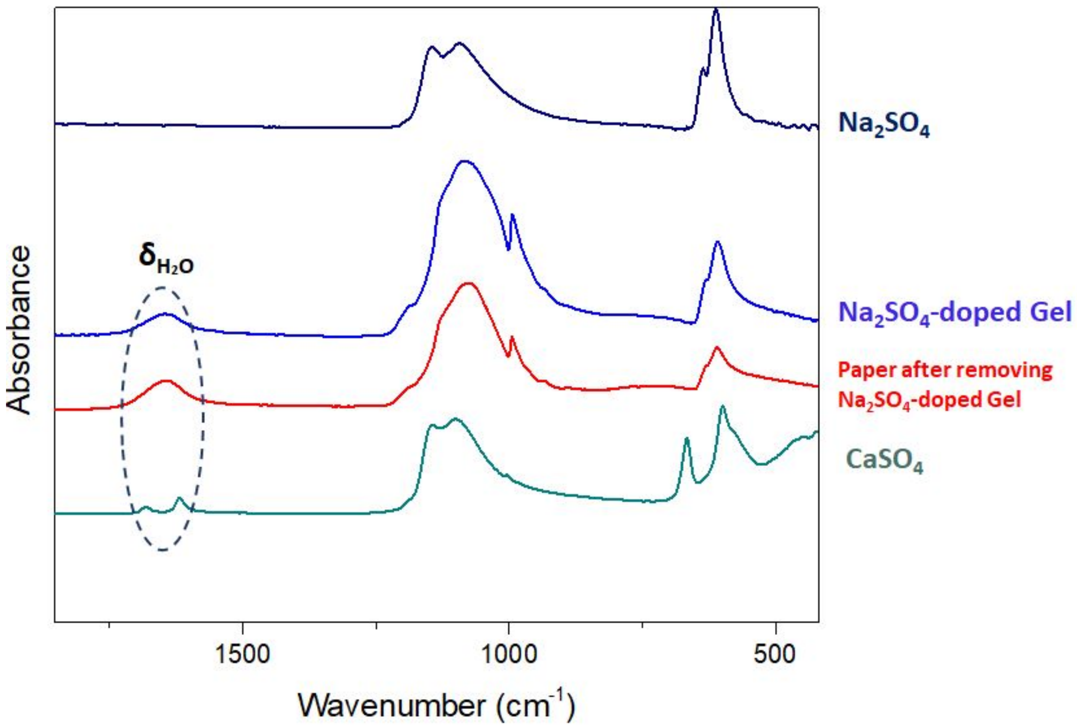

Figure S8. FTIR-ATR spectra of the Kraft paper after having contact with the hydrogel doped with Na_2SO_4 in comparison to the Na_2SO_4-doped gel, Na_2SO_4 powder, and $CaSO_4$ powder.

Note that the spectrum of the gel-contacted paper resembles that of Na_2SO_4 and differs from that of $CaSO_4$, indicating that calcium ions in the paper are not involved in the diffusion of sulfate ions.

X-ray fluorescence (XRF). The XRF analysis was done on a Rigaku ZSX Primus II instrument.

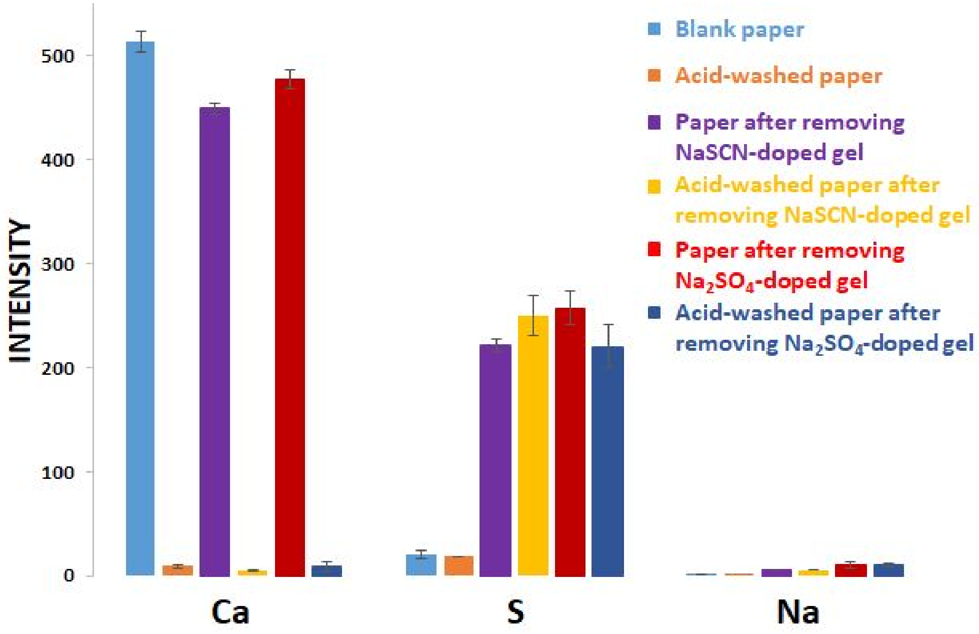

Figure S9. XRF data about the content of Ca, S, and Na in the blank Kraft paper, acid-washed Kraft paper, and those two after having contact with a hydrogel doped either with NaSCN or Na_2SO_4 salts. Another reason for the observed faster water diffusion with sulfate ions could be the calcium carbonate additive in the paper.[2] Calcium ions can form calcium sulfate with the sulfate, promoting water transport. XRF measurements of the interface on the blank paper and the acid-treated one (calcium carbonate removed)[3] dispute this hypothesis.

7. Mechanical properties of salt-doped gels (Young's modulus)

Dynamic mechanical analysis (DMA). The tensile and bending analyses were done on a TA Instruments DMA Q800 at the constant temperature of 55 ˚C.

The tensile test. The strain-induced was measured as a function of the tensile force with the 1 N/min force rate. Rectangular sheets of 40 mm x 15 mm x 2.5 mm were cut out of freshly prepared hydrogel bars (0.3 M salt and 5% agarose). Young's modulus for the tensile test was estimated in the linear elastic region of a material.

The bending test. A force rate of 1 N/min was performed on rectangular sheets of 35 mm x 15 mm x 2.5 mm cut out of freshly prepared hydrogel bars (0.3 M salt and 5% agarose). The bend allowance (BA) was calculated as:

$$BA = \frac{\theta\pi}{180}(R + 0.5T)$$

Where θ is the bending angle, R is the inner radius, and T is the thickness of the gel.

The compression test. The multi-frequency (1 Hz, 2.5 Hz, 5 Hz, 7.5 Hz, 10 Hz) DMA tests over the temperature range of 25 ˚C to 55 ˚C with a temperature ramp of 5 ˚C/min were performed on cubes of 10 mm x 10 mm x 4 mm (0.3 M salt and 5% agarose).

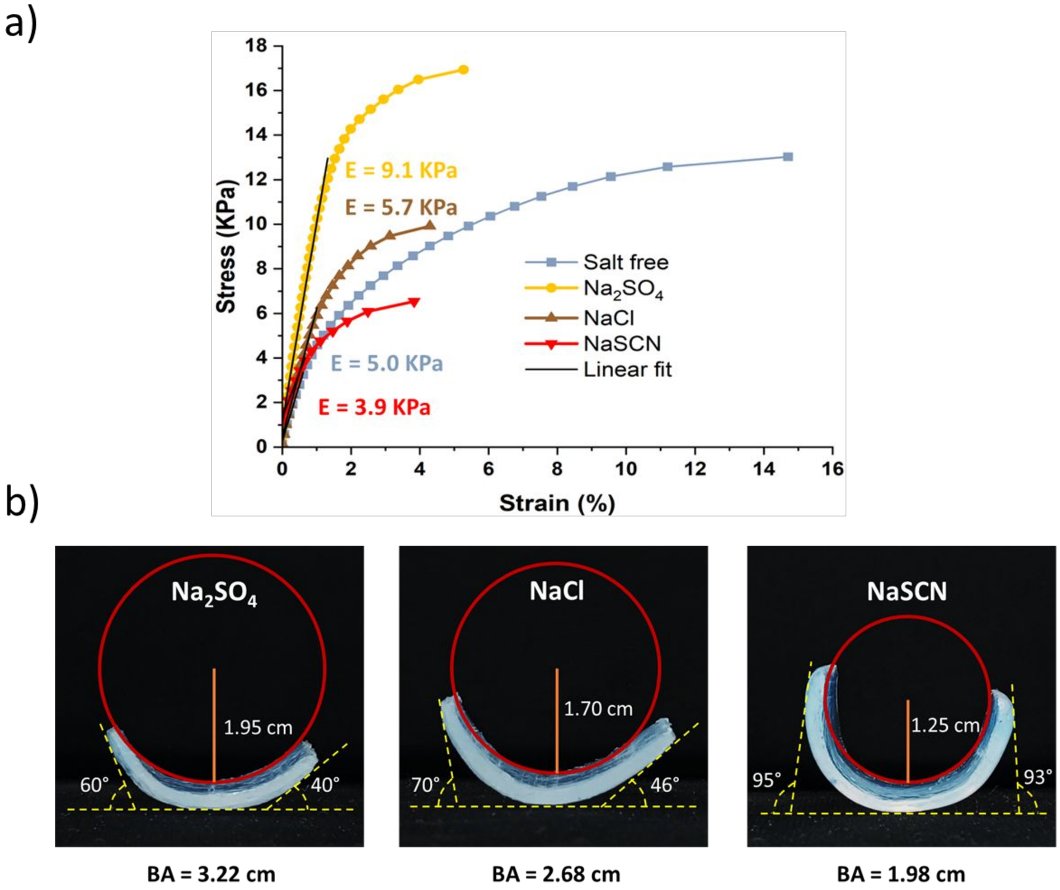

Figure S10. a) Stress versus the strain plots for the salt-free and salt-doped agarose hydrogels measured at 55 ˚C. b) The salt-doped gels after a 3-point bending test for 30 min at 55 ˚C.

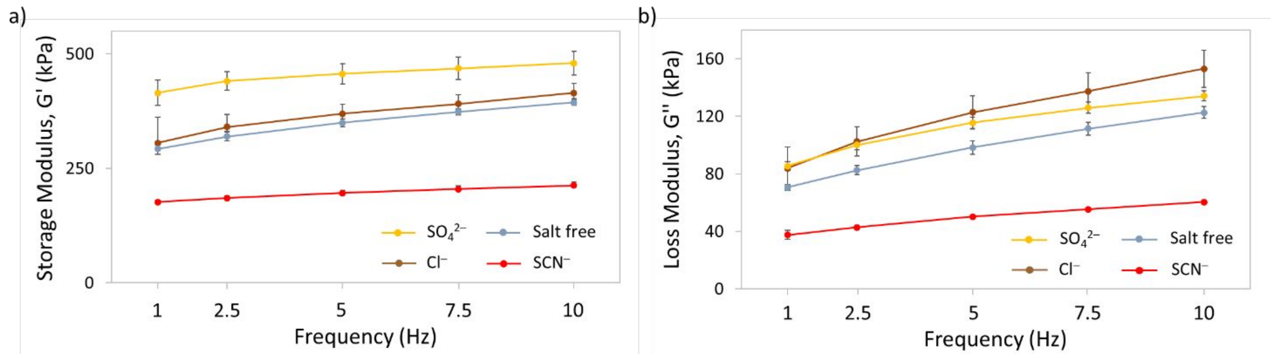

Figure S11. a) Storage modulus and b) loss modulus of salt-doped gels (0.3 M salt and 5% agarose). Sulfate-doped gel has a higher G′ and is therefore stiffer, thus bends less. It also has a higher G″ compared to that of thiocyanate-doped gel.

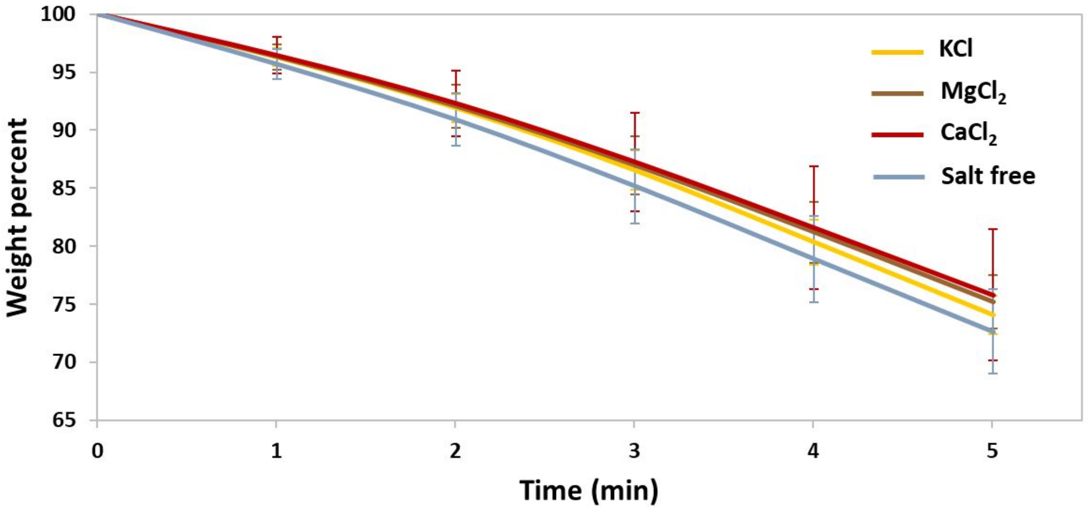

Figure S12. The TGA analysis of the chlorine salts (5.0 % agarose hydrogel, 0.30 M chlorine salts).

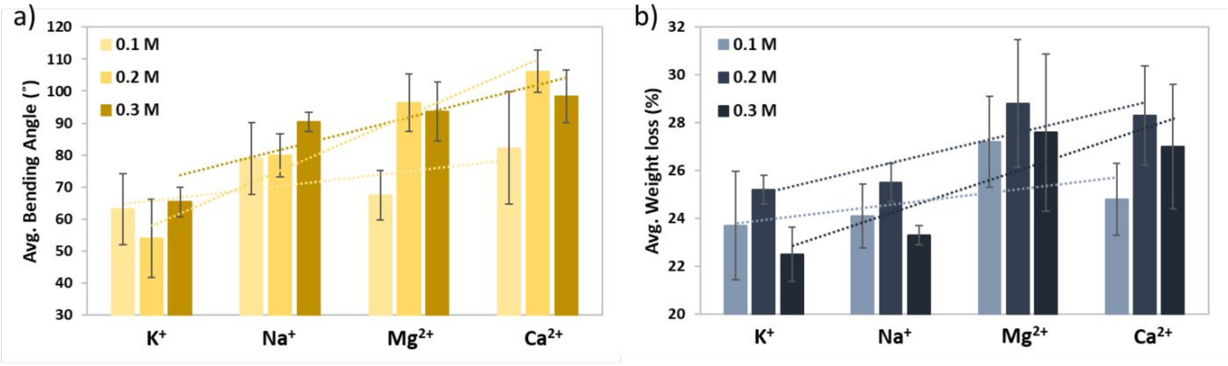

Figure S13. (a) The average bending angle and (b) the average weight loss of hydrogel actuators doped with chlorine-containing salts at different concentrations.

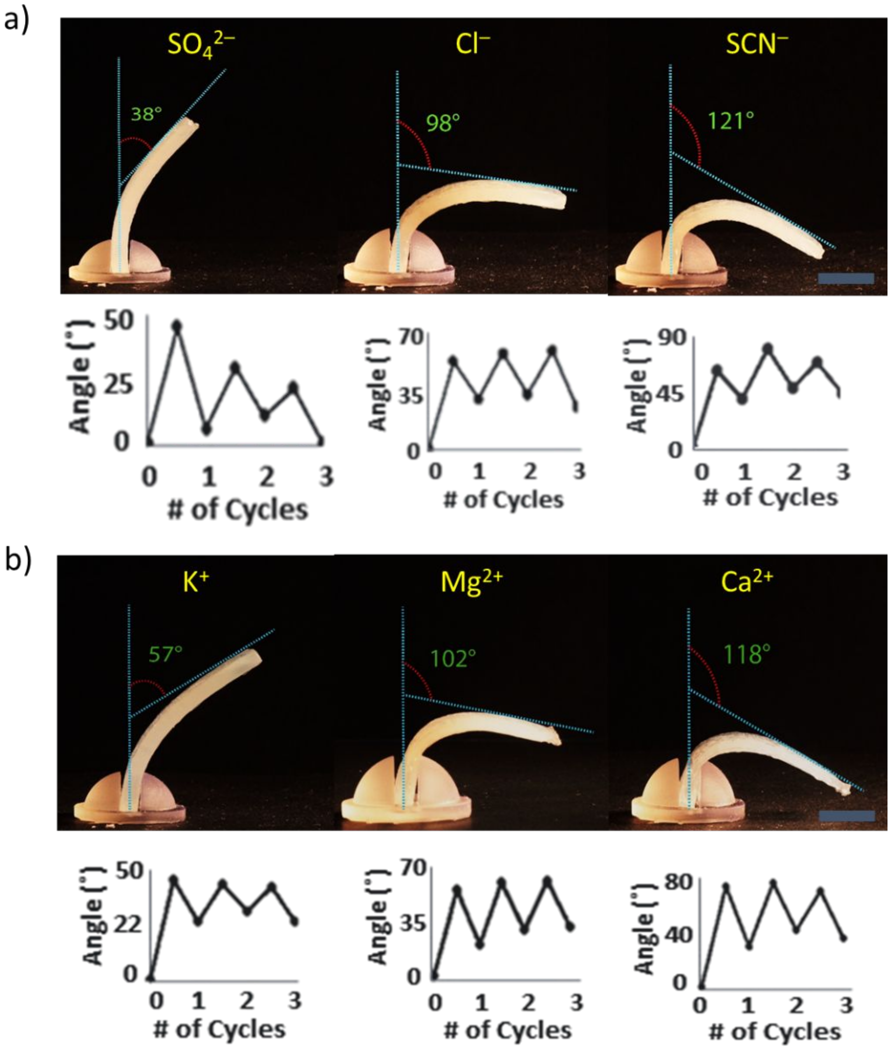

Figure S14. The reversibility test plots of the salty hydrogel actuators for (a) sodium salts and (b) chloride salts in three cycles. To prevent plastic deformation, the gels were not bent to the maximum bending. The gels were dehydrated for 20 min and rehydrated for 60 min in the first cycle. The subsequent cycles had shorter constant times, depending on the angle of bending.

8. Anisotropic actuation of the ‘salty’ hydrogel actuators

A salty pre-gel agarose solution of the salt, e.g., SO_4^{2-}, was poured into a 3D-printed mold with the desired geometry. After the gelation, half of the gel was cut and discarded from the mold, and the remaining space was filled with SCN^--doped pre-gel solution. Since agarose can crosslink physically, new bonds could form between the two halves when the newly poured half is gelated. The same procedure was followed for three different salts at three different parts of the sample, with an additional step for the third salt. The solutions were let gelate only for 15 min to minimize the diffusion of ions from one salty side to the other. The Raman spectra shown in Figure S14 show the diffusion extent of the ions in the samples to the neighboring sites after 15 min of gelation and 30 min of light illumination.

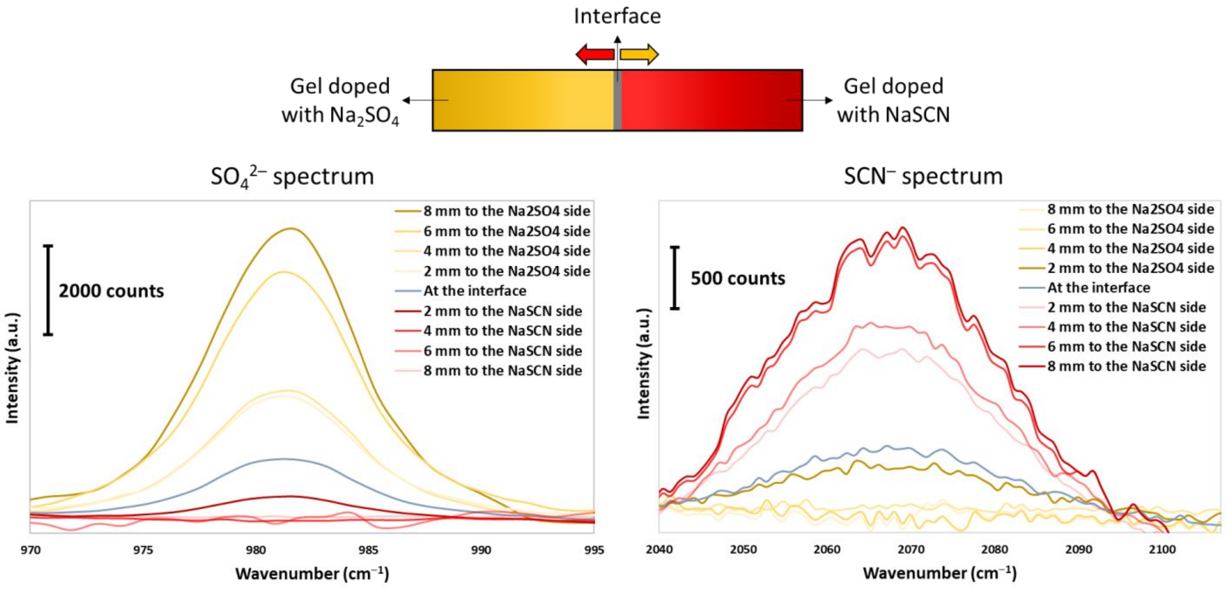

Figure S15. The Raman spectral analysis of sulfate and thiocyanate ions of the neighboring parts of a gel that has sulfate ions on one half and thiocyanate ions on the other, both of 0.30 M

concentration. The plots show the slow diffusion of ions from one side to the other after 30 minutes of light illumination.

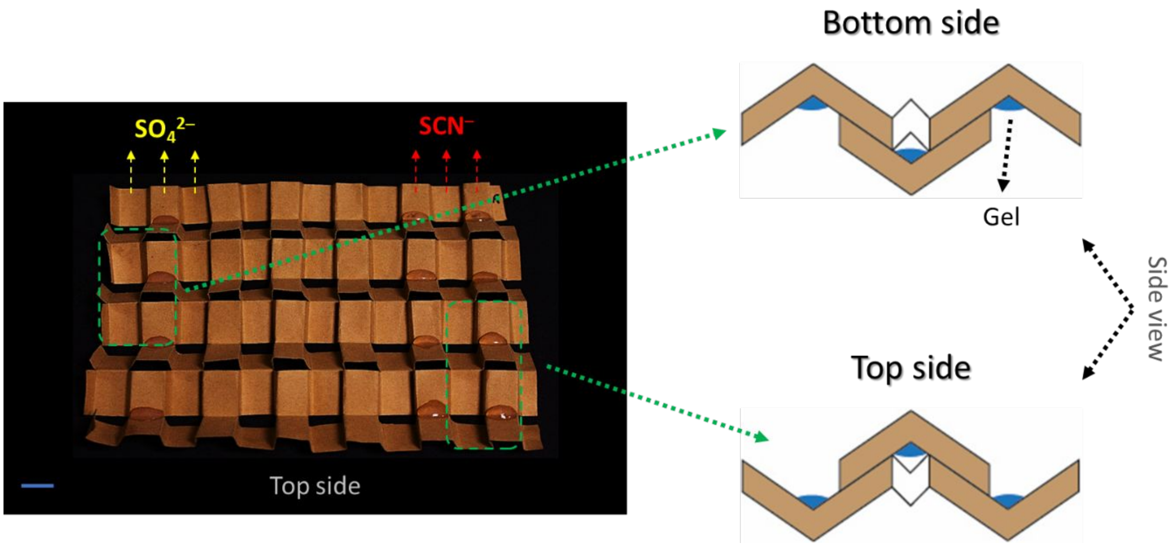

Figure S16. The kirigami design in Figure 6a, where the gels are added on the three consecutive crease rows of the paper. (Two rows on the top side and one row on the bottom for SCN^-, and vice versa for SO_4^{2-}).

References

(1) de Azevedo, A. R. G.; Alexandre, J.; Pessanha, L. S. P.; Manhães, R. da S. T.; de Brito, J.; Marvila, M. T. Characterizing the Paper Industry Sludge for Environmentally-Safe Disposal. *Waste Manag.* **2019**, *95*, 43–52. https://doi.org/10.1016/j.wasman.2019.06.001.

(2) Huang, X.; Shen, J.; Qian, X. Filler Modification for Papermaking with Starch/Oleic Acid Complexes with the Aid of Calcium Ions. *Carbohydr. Polym.* **2013**, *98* (1), 931–935. https://doi.org/10.1016/j.carbpol.2013.07.024.

(3) Ahn, E.; Kim, T.; Jeon, Y.; Kim, B. S. A4 Paper Chemistry: Synthesis of a Versatile and Chemically Modifiable Cellulose Membrane. *ACS Nano* **2020**, *14* (5), 6173–6180. https://doi.org/10.1021/acsnano.0c02211.
